# Enhanced Low-Temperature Hydrogen Storage in Nanoporous Ni-Based Alloy Supported LiBH_4_

**DOI:** 10.3389/fchem.2020.00283

**Published:** 2020-04-15

**Authors:** Xi Chen, Zhao Li, Yue Zhang, Dongming Liu, Chunyang Wang, Yongtao Li, Tingzhi Si, Qingan Zhang

**Affiliations:** School of Materials Science and Engineering, Anhui University of Technology, Maanshan, China

**Keywords:** hydrogen storage, lithium borohydride, nanoporous metal, nanoconfinement, catalysis

## Abstract

To reveal the synergistic effect of nanoconfinement and metallic catalysis on the hydrogen storage properties of LiBH_4_, the nanoporous Ni-based alloy (np-Ni) was prepared herein by dealloying of the Mn_70_Ni_30_ alloy in (NH_4_)_2_SO_4_ solution, and then LiBH_4_ was loaded into np-Ni to construct the LiBH_4_/np-Ni hydrogen storage system using wet impregnation. It was found that dehydrogenation of the LiBH_4_/np-Ni (1:5) system starts at around 70°C and ends before 400°C, with ~11.9 wt.% of hydrogen desorbed. The apparent dehydrogenation activation energy for the LiBH_4_/np-Ni (1:5) system was remarkable decreased to about 11.4 kJ/mol. After rehydrogenation at 450°C under 8 MPa hydrogen pressure, ~8.2 wt.% of hydrogen can be released from about 60°C upon second dehydrogenation. These obtained results would provide an efficient strategy for improving the hydrogen storage properties of other metal borohydrides.

## Introduction

Nowadays, the issue of energy shortage has been called into public focus. Hydrogen is considered to be the most ideal secondary source because of its high calorific value, low environmental impact and abundant resources (Abe et al., 2019). To meet the need of storing hydrogen with high efficiency and safety, it is necessary to develop hydrogen storage materials with high mass and volume hydrogen density (Yang et al., 2010; Li H. W. et al., 2011; Abdalla et al., 2018). LiBH_4_ has attracted much more attention due to its extremely high theoretical hydrogen storage capacity of 18.5 wt.%. However, the elevated dehydrogenation temperature, complicated dehydrogenation behavior and poor reversibility limit its practical applications (Züttel et al., 2003; Orimo et al., 2005; Mauron et al., 2008; Li C. et al., 2011). In order to overcome these deficiencies, the strategies of constructing reactive hydride system (Liu D. M. et al., 2013; Liu et al., 2015, 2016; Ding et al., 2019), cation/anion substitution (Yin et al., 2008; Fang et al., 2011), adding catalyst (Zhang et al., 2017; Cai et al., 2018) and nanoconfinement (Guo et al., 2017; Xu et al., 2017; Meng et al., 2018) were developed in the last decade.

Nanoconfinement of LiBH_4_ in nanoporous material can maintain the particle within a nanoscale structure, which is exceedingly beneficial to enlarge the reaction interface and shorten the element diffusion distance, thus significantly enhancing the de-/rehydrogenation properties (Ngene et al., 2010b; Shao et al., 2015; Meng et al., 2018; Gasnier et al., 2019). For example, Zhang et al. found that LiBH_4_ nanoparticles supported by disordered mesoporous carbon (CMK-3) showed a single dehydrogenation peak at about 332°C and a large dehydrogenation amount of 14 wt.% below 600°C (Zhang et al., 2007). Fang et al. embedded LiBH_4_ into active carbon (AC) by chemical impregnation. Due to the enhancement of both the thermodynamic and kinetic properties, the LiBH_4_/AC system began to release hydrogen at 220°C, which is 150°C lower than bulk LiBH_4_ without nanostructure modulation (Fang et al., 2008b). Other nanoporous material scaffolds, such as carbon aerogel (Zhao et al., 2014; Surrey et al., 2016), ordered mesoporous carbon (Cai et al., 2016), metallic organic framework (MOFs) (Sun et al., 2011) and mesoporous silicon dioxide (SBA-15) (Ngene et al., 2010a), were also used as the confinement carriers to support LiBH_4_.

However, the above reported nanoconfinement carriers are composed of non-metallic material that can only provide a single nanoconfinement role for LiBH_4_ in general. Taking into account that transition metal elements (e.g., Ni and Co) can serve as the active catalyst in improving the hydrogen storage properties of complex hydrides owing to their high electronegativity0 (Ngene et al., 2011; Liu et al., 2018; Zhang et al., 2018), a synergistic effect of nanoconfinement and catalysis would be achieved by confining LiBH_4_ in nanoporous transition metal. Based on this consideration, nanoporous Ni-based alloy was prepared by dealloying of the Mn_70_Ni_30_ alloy and then used as the carrier to support LiBH_4_ in this work, and a significantly improved low-temperature hydrogen storage in LiBH_4_ was successfully obtained.

## Experimental Section

### Sample Preparation

Commercial LiBH_4_ powder (95%, Alfa Aesar), Mn bulk (99.5%, Alfa Aesar), Ni sheet (99.5%, Alfa Aesar) and tetrahydrofuran (THF) (99.8%, anhydrous, Alfa Aesar) were used in experiments. The Mn_70_Ni_30_ alloy was prepared by induction melting of appropriate amounts of Mn and Ni metals. For compensating the loss of Mn during melting, the extra 3 wt.% of Mn was added on the basis of stoichiometric amount. The as-cast Mn_70_Ni_30_ alloy was mechanically crushed into powders of 200 mesh, and the nanoporous Ni-based alloy (denoted as np-Ni) was prepared by dealloying of the Mn_70_Ni_30_ alloy powders in 1 mol/L (NH_4_)_2_SO_4_ solution at 50°C for 2 h. The LiBH_4_/np-Ni (1:5) system was prepared by loading LiBH_4_ into np-Ni using wet impregnation method. Firstly, LiBH_4_ was dissolved in anhydrous THF. Then, np-Ni was put in the LiBH_4_ solution according to the LiBH_4_/np-Ni weight ratio of 1:5. Finally, the mixture was evacuated for 24 h to remove THF solvent.

### Sample Characterization

De-/rehydrogenation properties were examined based on the volumetric method by using a carefully calibrated Sieverts-type apparatus. Thermal dehydrogenation was performed by heating the sample from ambient temperature to 500°C at a rate of 2°C/min. Isothermal dehydogenation was performed by quickly heating and then keeping the sample at a given temperature. The hydrogen back pressure for the above temperature ramp and isothermal dehydrogenation examinations was below 0.1 MPa. Isothermal rehydrogenation was carried out at 450°C under 8 MPa hydrogen pressure. The weight of np-Ni was not taken into account in calculating the hydrogen de-/absorption amounts.

X-ray diffraction (XRD) measurement was performed by a Rigaku D/Max 2500VL/PC diffractometer at 50 kV and 200 mA with Cu Kα radiation. A special Ar-filled holder was applied to seal the XRD sample to avoid contact with air in the course of measurement. To quantitatively investigate the phase structure change of the Mn_70_Ni_30_ alloy before and after dealloying, the XRD profiles were analyzed with the Rietveld refinement program RIETAN-2000 (Izumi and Ikeda, 2000). Scanning electron microscopy (SEM) was carried out using a Nova NanoSEM 430 microscope equipped with an energy dispersive X-ray spectrometer (EDS). Transmission electron microscopy (TEM) observation was performed on a JEM-2100F instrument. Pore size distribution, pore volume and specific surface area were determined by a Micromeritics ASAP 2020 fully-automatic analyzer based on the Brunauer–Emmett–Teller (BET) and Barrett–Joyner–Halenda (BJH) methods (Lowell et al., 2004). Fourier transform infrared (FTIR) spectrum was collected using a Nicolet 6700 FTIR spectrometer.

## Results and Discussion

### Structural Analysis of LiBH_4_/np-Ni System

**Figure 1:**
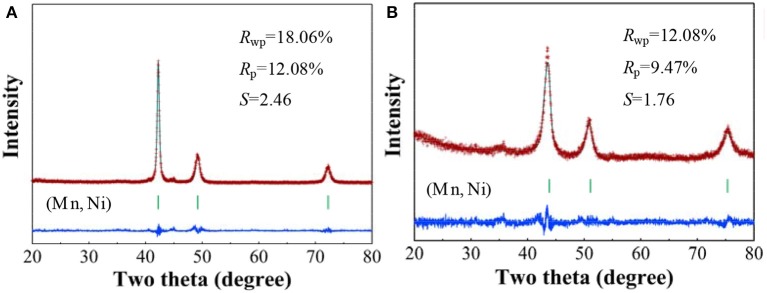
Rietveld refinements of the observed XRD patterns for Mn_70_Ni_30_ alloy **(A)** before and **(B)** after dealloying.

Figure 1 gives the observed XRD patterns and the Rietveld analysis results of Mn_70_Ni_30_ alloy before and after dealloying, and the phase abundances and structural parameters refined by the Rietveld analysis are listed in Table 1. It can be seen that the Mn_70_Ni_30_ alloy before and after dealloying are both composed of a single phase of (Mn, Ni) solid solution with a Cu-type structure. However, the XRD peaks of np-Ni are relatively broadened and move toward higher angle as compared with the Mn_70_Ni_30_ alloy. The results indicate that the grain size and cell parameters of the sample were both decreased with the extraction of Mn atom from (Mn, Ni) solid solution upon dealloying due to that Mn has a larger atomic radius relative to Ni.

**Table 1:** Phase components and structural parameters of Mn_70_Ni_30_ alloy and np-Ni.

**Sample**	**Phase**	**Space group**	**Lattice parameters (Å)**			**Abundance (%)**
			***a***	***b***	***c***	
Mn_70_Ni_30_ alloy	(Mn, Ni)	*Fm-3m*	3.6907(2)	3.6907(2)	3.6907(2)	100
np-Ni	(Mn, Ni)	*Fm-3m*	3.5601(1)	3.5601(1)	3.5601(1)	100

**Figure 2:**
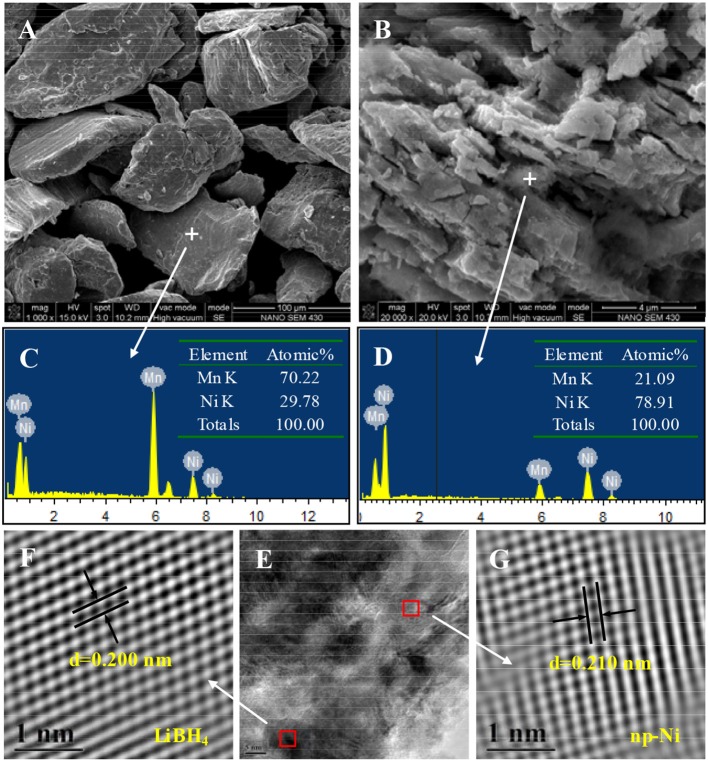
SEM images and EDS spectra of **(A,C)** Mn_70_Ni_30_ alloy and **(B,D)** np-Ni; **(E)** TEM micrograph of the LiBH_4_/np-Ni (1:5) system and **(F,G)** atomic lattice images of the square regions in **(E)**.

Figure 2 presents the SEM images and corresponding EDS spectra of Mn_70_Ni_30_ alloy and np-Ni. As seen from Figure 2A, the Mn_70_Ni_30_ alloy has a smooth surface with a particle size of about ~70 μm. The EDS result (see Figure 2C) indicates that it consists of 70.22 at.% Mn and 29.78 at.% Ni, agreeing well with its nominal element composition. For np-Ni, as given in Figure 2D, the element content of Mn is decreased to 21.09 at.%. It is reasonably considered that the massive lixiviation of Mn atom can bring large lattice distortion and physical shrinkage stress, thus leading to the formation of a nanoporous structure as shown in Figure 2B.

**Figure 3:**
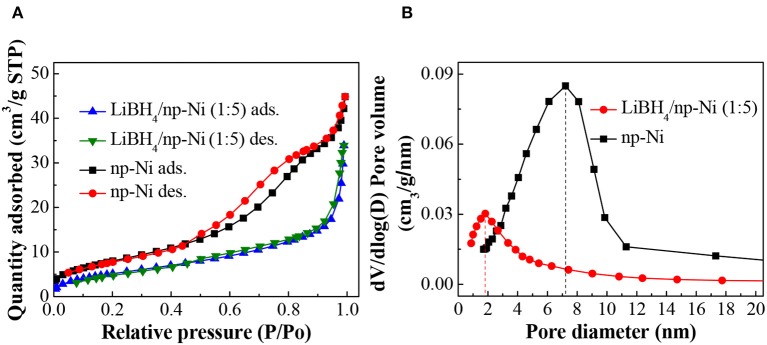
**(A)** N_2_ adsorption/desorption isotherms and **(B)** pore size distributions of np-Ni and the LiBH_4_/np-Ni (1:5) system.

Figure 3A demonstrates the N_2_ adsorption/desorption isotherms for np-Ni and the LiBH_4_/np-Ni (1:5) system. It can be seen that np-Ni has a typical IV-type adsorption isotherm with an obvious hysteresis loop. Those are the characteristics of mesoporous material. In comparison, the hysteresis loop has almost disappeared for the LiBH_4_/np-Ni (1:5) system. The pore size distributions of np-Ni and the LiBH_4_/np-Ni (1:5) system are compared in Figure 3B, which indicates that the peak in pore size distribution of np-Ni moves to a lower position with an intensive decline in intensity after supporting LiBH_4_. Table 2 gives the pore parameters and specific surface area of np-Ni and the LiBH_4_/np-Ni (1:5) system. It is observed that np-Ni has the pore diameter, pore volume and specific surface area of 7.21 nm, 0.0586 cm^3^/g and 155 m^2^/g, respectively. However, those values reduce to 1.80 nm, 0.0339 cm^3^/g and 17 m^2^/g, respectively, for the LiBH_4_/np-Ni (1:5) system. These results imply that LiBH_4_ was loaded on the surface and impregnated into the pores of np-Ni. Figure 2E gives the TEM micrograph of the LiBH_4_/np-Ni (1:5) system, and Figures 2F,G present the atomic lattice images of the square regions in Figure 2E obtained by inverse fast Fourier transform (IFFT). The fringe spacings of 0.200 nm in Figure 2F and 0.210 nm in Figure 2G correspond to (121) plane of LiBH_4_ and (111) plane of Ni, respectively. The TEM results reveal that LiBH_4_ and np-Ni indeed co-existed in the sample.

**Table 2:** Pore parameters and specific surface area of np-Ni and the LiBH_4_/np-Ni (1:5) system.

**Sample**	**Pore size (nm)**	**Pore volume (cm^**3**^/g)**	**Specific surface area (m^**2**^/g)**
np-Ni	7.21	0.0586	155
LiBH_4_/np-Ni	1.80	0.0339	17

### Thermal Dehydrogenation Characteristics of LiBH_4_/np-Ni System

**Figure 4:**
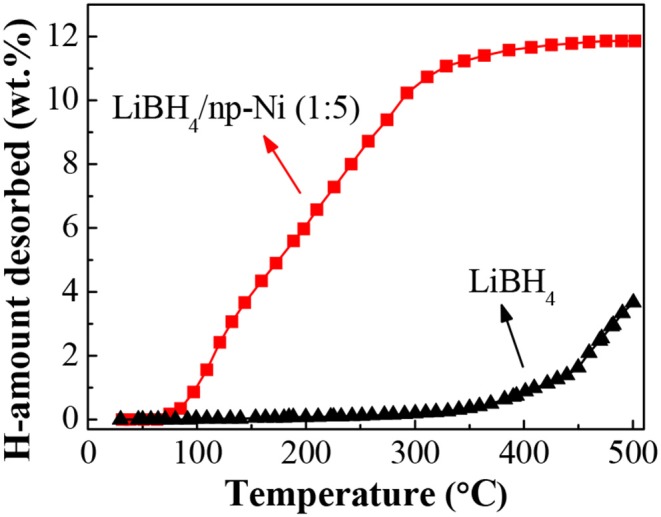
Hydrogen desorption curves of the LiBH_4_/np-Ni (1:5) system and pristine LiBH_4_.

Figure 4 shows the temperature-programmed dehydrogenation curves of the LiBH_4_/np-Ni (1:5) system and pristine LiBH_4_. It can be seen that hydrogen release from the LiBH_4_/np-Ni (1:5) system initiates at about 70°C and ends before 400°C, with ~11.9 wt.% of hydrogen desorbed. In contrast, the starting dehydrogenation temperature is as high as 330°C for pristine LiBH_4_, and only 3.5 wt.% of hydrogen can be released when heating to 500°C. Evidently, the thermal dehydrogenation stability of LiBH_4_ was notably reduced by np-Ni. In addition, Table 3 compares the dehydrogenation temperature of LiBH_4_ supported on different carriers. It is observed that the present LiBH_4_/np-Ni (1:5) system has lower starting and ending dehydrogenation temperatures as compared with the reported LiBH_4_-based supporting systems. In other words, np-Ni can provide a stronger destabilization effect on LiBH_4_ relative to other carriers due to its synergistic effect of nanoconfinement and metallic catalysis. On the one hand, nanoconfinement of LiBH_4_ in np-Ni can decrease the particle size to nanoscale level, which is very helpful to facilitate the dehydrogenation by enlarging the reaction interface and shortening the element diffusion distance. On the other hand, Ni itself can act as the dehydrogenation catalyst for LiBH_4_ by enhancing charge donation ability of Li atom to BH_4_ unit and thus weakening the B–H bond due to its high electronegativity.

**Table 3:** Hydrogen desorption temperature of LiBH_4_ with different carriers.

**Carriers**	**Starting temperature (°C)**	**Ending temperature (°C)**	**References**
ZnO/ZnCo_2_O_4_	169	<500	Xu et al., 2017
CMK-3	220	<600	Zhang et al., 2007
Carbon aerogels@CoNiB	192	600	Zhao et al., 2014
SBA-15	150	>500	Ngene et al., 2010a
Single-walled carbon nanotubes	270	550	Fang et al., 2008a
Carbon nanotubes	250	<600	Yu et al., 2007
Nanoporous carbon	220	420	Liu et al., 2010
Nanoscale SiO_2_	200	500	Chen et al., 2010
np-Ni	70	400	This work

**Figure 5:**
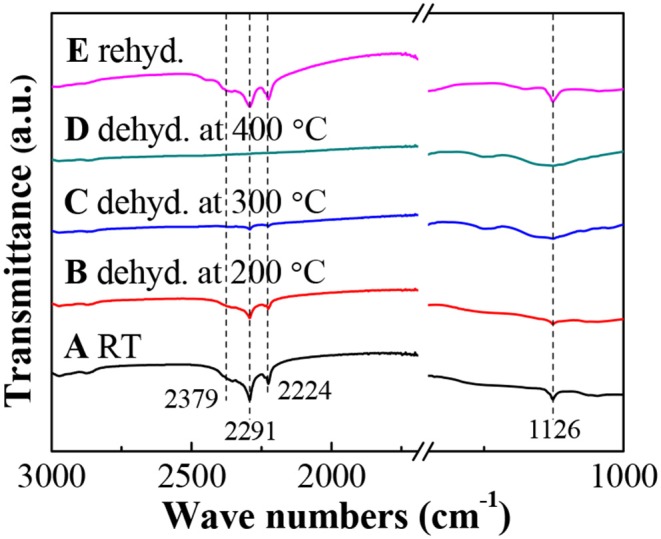
FTIR spectra of the LiBH_4_/np-Ni (1:5) system dehydrogenated at different temperatures and rehydrogenated.

To further monitor the dehydrogenation process, Figure 5 gives the FTIR spectra of LiBH_4_/np-Ni (1:5) systems after dehydrogenation at different temperatures. As can be seen in Figure 5A, the obvious characteristic bands for B–H bond vibrations located at 2,379, 2,291, 2,224 and 1,126 cm^−1^ (Zhang et al., 2011) confirm the existence of LiBH_4_. With increasing the dehydrogenation temperature, the band intensity of B–H bond vibrations decreases gradually, indicating a continuous consumption of LiBH_4_. Moreover, almost no FTIR bands can be observed in Figure 5D, which means that LiBH_4_ was almost completely decomposed at 400°C. This result is in good agreement with the dehydrogenation phenomenon shown in Figure 4.

### Dehydrogenation Kinetics of LiBH_4_/np-Ni Systetm

**Figure 6:**
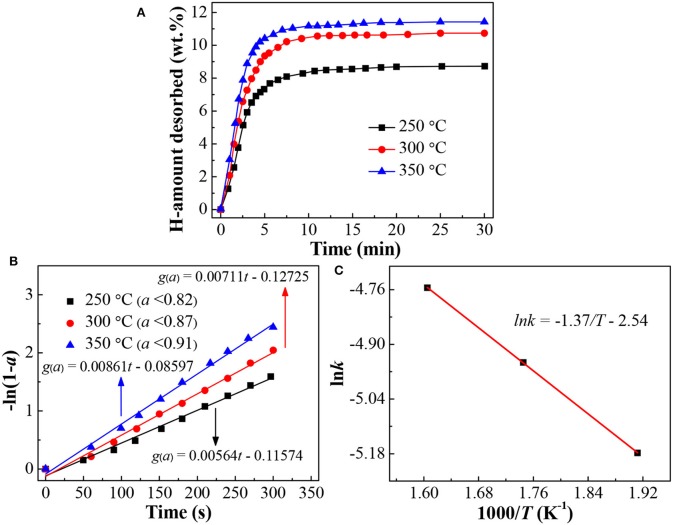
**(A)** Isothermal dehydrogenation curves, **(B)** Plots of -ln(1-*a*) vs. *t* at different temperatures, and **(C)** Arrhenius plot for the dehydrogenation of the LiBH_4_/np-Ni (1:5) system.

Figure 6A presents the isothermal dehydrogenation curves of the LiBH_4_/np-Ni (1:5) system at the temperatures of 250, 300, and 350°C, respectively. It is observed that the dehydrogenation rate increases as the temperature rises. For example, the amounts of hydrogen desorbed within 5 min are 7.3, 9.4, and 10.4 wt.% at 250, 300, and 350°C, respectively. In order to further reveal the dehydrogenation mechanism, the experimental dehydrogenation data were fitted by the kinetic modeling of *g*(α) = ∫*d*α/*f* (α) = *kt*, where α is the reacted fraction at time *t, g*(α) and *f* (α) are the functions representing different reaction mechanisms, and *k* is the rate constant (Li Y. et al., 2011; Liu D. M. et al., 2013). As the result, the function of -ln(1*-*α) gives the best linearity (see Figure 6B) over a broader α range for each measurement with the correlation coefficient of *R*^2^ >0.99. This result indicates that dehydrogenation of the LiBH_4_/np-Ni (1:5) system follows the first-order mechanism in the investigated temperature range.

According to the slope of the fitted straight line in Figure 6B, the *k* value at different temperatures can be obtained. Then the apparent activation energy for hydrogen desorption (*E*_a_) can be determined based on the Arrhenius equation of *k* = *k*_0_ · exp[-*E*_a_/(*RT*)], where *k*_0_ is the pre-exponential factor, *R* is the gas constant, and *T* is the temperature. Figure 6C gives the Arrhenius plot for the LiBH_4_/np-Ni (1:5) system. From the slope (-*E*_a_/*R*) of the fitted straight line, *E*_a_ was calculated to be 11.4 kJ/mol. As reported in the literatures that *E*_a_ for LiBH_4_ supported on CMK-3 and carbon aerogels@CoNiB are 40 and 46.39 kJ/mol, respectively (Zhang et al., 2007; Zhao et al., 2014). The lower *E*_a_ value for the present LiBH_4_/np-Ni (1:5) system is originating from the synergistic effect of nanoconfinement and metallic catalysis of np-Ni, and can be regarded as one of the most important reasons for the enhanced dehydrogenation properties shown in Figures 4, 6A. Moreover, the preparation process of np-Ni carrier for LiBH_4_ by dealloying method is far more convenient than that of CMK-3 based on template method.

### Rehydrogenation Characteristics of LiBH_4_/np-Ni System

**Figure 7:**
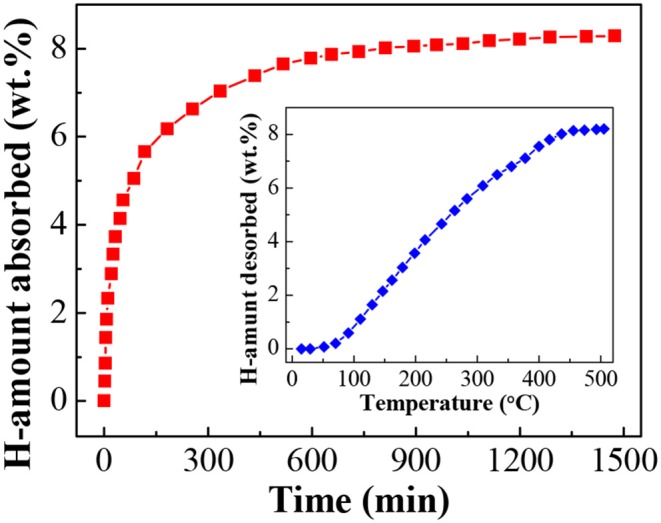
Isothermal rehydrogenation curve of the LiBH_4_/np-Ni (1:5) system. Inset shows the second hydrogen desorption curve.

The dehydrogenated residue of the LiBH_4_/np-Ni (1:5) system was subjected to rehydrogenation, and Figure 7 demonstrates the isothermal rehydrogenation curve. It is observed that the LiBH_4_/np-Ni (1:5) system can readily reabsorb 8.3 wt.% of hydrogen at 450°C under 8 MPa hydrogen pressure. The FTIR spectrum for the rehydrogenated product shown in Figure 5E suggests that LiBH_4_ was regenerated. The inset of Figure 7 gives the second hydrogen desorption curve of the LiBH_4_/np-Ni (1:5) system. It can be seen that ~8.2 wt.% of hydrogen can be released during the second dehydrogenation process. Note that the starting dehydrogenation temperature keeps in a low value of about 60°C. The result indicates undoubtedly that the synergistic effect of nanoconfinement and metallic catalysis of np-Ni maintains well upon repeated dehydrogenation/hydrogenation.

## Conclusions

In order to improve the hydrogen storage properties of LiBH_4_, the nanoporous Ni-based alloy was prepared by dealloying of the precursor Mn_70_Ni_30_ alloy and then used as the carrier to support LiBH_4_ by wet impregnation method. It was found that the constructed LiBH_4_/np-Ni (1:5) system can release ~11.9 wt.% of hydrogen with the starting and ending dehydrogenation temperatures as low as about 70 and 400°C, respectively. Due to the synergistic effect of nanoconfinement and metallic catalysis of nanoporous Ni-based alloy, the apparent dehydrogenation activation energy of LiBH_4_ was remarkable decreased to about 11.4 kJ/mol. The dehydrogenated residue can readily absorb hydrogen to regenerate LiBH_4_ at 450°C under 8 MPa hydrogen pressure. Moreover, the starting dehydrogenation temperature keeps in a low value of about 60°C during the second dehydrogenation process.

## Data Availability Statement

All datasets generated for this study are included in the article.

## Author Contributions

XC and DL contributed conception and design of the study. XC and YZ were in charge of the analysis of data. ZL and CW prepared samples and performed characterization. All authors contributed to manuscript revision, read, and approved the submitted version.

### Conflict of Interest

The authors declare that the research was conducted in the absence of any commercial or financial relationships that could be construed as a potential conflict of interest.
